# An Artificial Intelligence Approach to Predict Tracheostomy Requirement in Mechanically Ventilated Critically Ill Patients: A Retrospective Single-Center Study

**DOI:** 10.3390/jcm15052081

**Published:** 2026-03-09

**Authors:** Dicle Birtane, Fatma Özdemir, Damla Yavuz, Zafer Çukurova

**Affiliations:** 1 Department of Anesthesiology and Reanimation Intensive Care, Bakirkoy Dr. Sadi Konuk Training and Research Hospital, 34000 Istanbul, Turkey; fatmataskin88@hotmail.com (F.Ö.); zcukurova@gmail.com (Z.Ç.); 2 Department of Anesthesiology and Reanimation, Denizli Government Hospital, 20000 Denizli, Turkey; yavuzdamla1990@gmail.com

**Keywords:** artificial intelligence, intensive care unit, machine learning, prediction, secretion, tracheostomy

## Abstract

**Background:** In critically ill patients, tracheostomy decisions are driven by heterogeneous and dynamic clinical trajectories, and no universally accepted scoring system exists to reliably predict tracheostomy requirement. An accurate and interpretable prediction model could help earlier decision-making and potentially reduce prolonged mechanical ventilation (MV) and failed weaning. **Methods:** In this retrospective study, data from 6507 mechanically ventilated intensive care unit (ICU) patients were analyzed using an electronic clinical decision support system; 1049 patients required tracheostomy and 5458 did not. The primary outcome was the prediction of tracheostomy occurrence during ICU stay based on invasive mechanical ventilation (IMV) parameters obtained within the first five days. The secondary outcome was the identification of the most influential parameters guiding tracheostomy decision-making during early IMV. Ten machine learning algorithms were developed using an 80/20 train–test split. Model performance was assessed using discrimination, calibration, and clinical performance metrics. Explainability was evaluated using SHapley Additive exPlanations (SHAP) analysis. **Results:** Among all models, Gradient Boosting demonstrated strong discrimination and calibration performance (AUROC 0.92, AUPRC 0.56, specificity 97%, F1 score 0.46, Brier score 0.078). In the Gradient Boosting model, feature importance analysis demonstrated that secretion count was the strongest predictor of tracheostomy requirement, accounting for 14.72% of the model’s predictive contribution. This was followed by lactate level (6.12%), arterial pH (3.74%), and peak airway pressure (3.57%). SHAP-based analyses consistently identified secretion count as the strongest predictor of tracheostomy requirement, followed by lactate level, Glasgow Coma Scale (GCS), and arterial pH. In addition, SHAP provided clinically interpretable insights into the direction and magnitude of the effects of individual predictors. **Conclusions:** Machine learning models integrating early-phase ventilatory and physiological data may enable clinically meaningful prediction of tracheostomy requirement. The combination of strong performance and explainability suggests potential utility as a decision-support tool in critically ill patients requiring prolonged MV.

## 1. Introduction

Among critically ill patients in the intensive care unit (ICU), difficulties in discontinuing mechanical ventilation (MV) can arise from multiple factors. In such situations—especially in patients requiring prolonged ventilatory support, those with traumatic brain injury, or individuals with neurological conditions impairing airway protection—tracheostomy is frequently undertaken. Tracheostomy may provide several potential benefits, including facilitating weaning from MV, reducing sedation requirements, improving patient comfort, and shortening ICU length of stay; additionally, it can serve as a route for the administration of inhaled medications [1,2,3]. But the optimal timing of the procedure remains unclear. Tracheostomy is also associated with serious complications, including pneumothorax, bleeding, subglottic stenosis, fistula formation, stoma infection, and dysphonia [4,5]. Time and appropriate patient selection are crucial; early tracheostomy (performed < 10 days) was associated with shorter hospitalization, faster transition to noninvasive ventilation, and a shorter duration of MV [6]. A meta-analysis of 21 randomized controlled trials demonstrated that early tracheostomy significantly reduced the duration of MV, while no significant effect on mortality was observed [7]. A meta-analysis including nine studies comparing early and late tracheostomy, using a 10-day threshold in patients without neurological injury, demonstrated comparable mortality rates between the two groups [8]. In an analysis of eight randomized controlled trials comparing three different tracheostomy timing intervals, the ≤4-day group was found to be associated with lower short-term mortality compared with the ≥13-day group [9]. Its timing varies according to the patient’s clinical status and the clinician’s judgment, and no standardized approach currently exists. International guidelines provide only weak recommendations regarding tracheostomy timing due to substantial heterogeneity in patient populations, marked variability in clinical practice, and the absence of reliable predictive tools. Early tracheostomy appears to offer some benefits across clinical outcomes, but systematic reviews have highlighted inconsistent findings and lack of definitive evidence to support a single standardized timing approach [10].

Accurately predicting the need for tracheostomy at an early stage is critically important for clinical management. Timely recognition of patients who are likely to need tracheostomy may help avoid unnecessarily prolonged MV and excessive sedation, whereas delayed identification can increase the risk of ventilator-related injury and potentially promote diaphragmatic dysfunction. Identifying patients who would benefit from tracheostomy also improves the efficient use of healthcare resources and prevents unnecessary interventions. Considering the reality of limited resources and time, the need for individualized and optimal treatment strategies is evident. Moreover, improving our ability to predict tracheostomy requirements enhances communication with patients and their families, thereby improving satisfaction and adherence to treatment. Unfortunately, there is currently no advanced, universally applicable, and generalizable predictive scale or classification system that can be used objectively to guide decision-making.

Advances in artificial intelligence (AI) technologies enable real-time monitoring of patients and facilitate the implementation of individualized ventilation management strategies [11]. Machine learning (ML) methods outperform traditional statistical models in critically ill patients by evaluating complex and non-linear interactions among multiple parameters rather than relying on a single variable or system. In predicting prolonged MV and the need for tracheostomy, studies using ML have demonstrated that pulmonary characteristics constitute the most influential parameters, whereas cardiac arrhythmia is the most significant comorbidity associated with these outcomes [12]. Likewise, a study of patients with deep neck infections demonstrated that an AI system built on deep learning techniques could effectively predict the need for tracheostomy [13]. In recent years, ML has shown considerable success in predicting prolonged MV and extubation failure; however, studies aimed at forecasting tracheostomy requirements remain limited. Most existing models rely on variables obtained at the time of ICU admission, overlooking the impact of changes in clinical and physiological parameters over longer periods. Yet, such longitudinal variability may carry substantial predictive value.

This study sought to develop and validate a ML model capable of predicting the need for tracheostomy by integrating the physiological parameters and clinical characteristics of patients who required at least five days of invasive mechanical ventilation (IMV) from the time of ICU admission. In addition, we aimed to perform variable importance analyses to enhance the interpretability of the model for clinicians.

## 2. Materials and Methods

### 2.1. Study Design and Population

**Figure 1:**
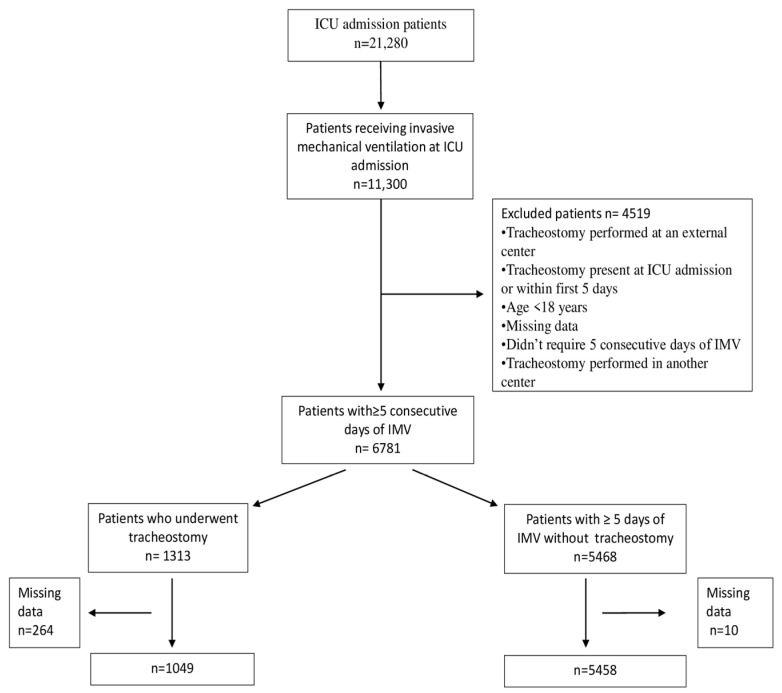
Flowchart of the study patients.

A retrospective, single-center study was conducted in the general ICU of a tertiary hospital between 1 January 2015 and 1 January 2025. Adult patients (>18 years) who required IMV for at least 5 consecutive days after admission to the ICU were included. The primary outcome was the prediction of tracheostomy during ICU stay based on IMV parameters obtained within the first five days. The secondary outcome was the identification of the most influential parameters guiding tracheostomy decision-making during early IMV. Patients who had a tracheostomy at the time of ICU admission, those younger than 18 years, individuals with missing data, patients who did not require five consecutive days of IMV, and those who underwent tracheostomy in another center were excluded. The research protocol was reviewed and approved by the institutional ethics committee (Approval No: 2025-02-19; Date: 7 November 2025). All procedures adhered to relevant ethical standards and regulations, including the Declaration of Helsinki. Clinically implausible values were treated as erroneous and excluded. Missing data were not imputed, and analyses were performed using complete-case records only (Figure 1).

### 2.2. Data Collection

All data were retrospectively extracted from the ImdSoft—Metavision/QlinICU Clinical Decision Support System(Canada) using structured SQL queries. Minute-by-minute data were aggregated into hourly means and subsequently averaged to obtain daily values.

### 2.3. Evaluated Parameters

#### 2.3.1. Demographic and Baseline Clinical Data

Age, sex, predicted body weight, body mass index (BMI), comorbidities, and Acute Physiology and Chronic Health Evaluation II (APACHE II) scores recorded within the first 24 h of ICU admission were analyzed. Primary diagnoses were categorized systematically.

#### 2.3.2. Vital Signs and Secretion Count

Vital signs, including body temperature, heart rate (HR), systolic, diastolic, and mean arterial blood pressure, as well as respiratory rate (RR), were converted from minute-by-minute recordings into hourly averages and subsequently into daily mean values. The count of secretion aspirations performed during the first five days of IMV was recorded.

#### 2.3.3. Neurological and Organ Dysfunction Scores

Glasgow Coma Scale (GCS) (modified GCS due to invasive ventilation) [14] and Sequential Organ Failure Assessment (SOFA) scores were recorded daily during the first five days of IMV. HACOR scores (Heart rate, Acidosis, Consciousness, Oxygenation, Respiratory Rate) were calculated using daily averaged component values according to established criteria [15].

#### 2.3.4. Respiratory Mechanics, Gas Exchange, and Laboratory Parameters

Respiratory mechanics, including mean mechanical power (MP), ventilator parameters, including tidal volume (VT), positive end-expiratory pressure (PEEP), peak inspiratory pressure (PPeak), driving pressure (ΔP), dynamic compliance (CompD) (and volume oxygenation index (VOX), were analyzed during the first five days of IMV using electronically recorded data aggregated into daily mean values. MP was calculated as VT × (PPeak − ΔP/2) × RR × 0.098 [16]. VOX was calculated as (SpO_2_/FiO_2_) × VT [17]. Arterial blood gas parameters (pH, PaO_2_, PaCO_2_, lactate), derived indices including oxygenation index (OI) and oxygen saturation index (OSI), and laboratory variables (potassium, magnesium, albumin, procalcitonin, and C-reactive protein) were also evaluated. OI and OSI were calculated as (FiO_2_ × mean airway pressure × 100)/PaO_2_ and /SpO_2_, respectively [18].

### 2.4. Model Development

#### 2.4.1. Feature Engineering and Model Input

Variables included in the ML models comprised demographic characteristics and ICU- and admission-related features considered potentially associated with tracheostomy requirement. Categorical variables were encoded using one-hot encoding, and continuous variables were normalized using min–max scaling. Variables with more than 10% missing data and variables representing post-tracheostomy events were excluded from the standardization process. Variable selection was guided by a comprehensive literature review, clinical judgment regarding factors associated with tracheostomy requirement, and exploratory analysis of the overall dataset.

The dataset was initially randomly divided into training (80%) and test (20%) sets to enable an unbiased evaluation of real-world model performance. The test set was strictly held out during model development and was used exclusively for final performance evaluation.

To enhance robustness during model development, 5-fold stratified cross-validation was applied within the training set. Specifically, the training set was partitioned into five equal folds. In each iteration, four folds were used for model training and the remaining fold for validation. This procedure was repeated five times so that each fold served as a validation set once.

For each model, performance metrics—including accuracy, Receiver Operating Characteristic Curve–Area Under the Curve (ROC–AUC) and Area Under the Precision–Recall Curve (AUPRC), sensitivity, specificity, F1-score, and Brier score—were calculated within each fold. The mean and standard deviation across the five folds were computed to assess model stability during development.

Model selection was based on cross-validation performance within the training set to enhance stability and mitigate the risk of overfitting.

The final selected model was subsequently evaluated on the independent test dataset. Test-set performance metrics were reported as the primary performance estimates. Calibration on the independent test dataset was assessed using calibration curves, calibration slope, calibration intercept, and Brier score.

#### 2.4.2. Definition of Tracheostomy and Group Classification

Within this cohort, tracheostomy status was determined by querying records related to treatment, daily follow-up, monitoring, events, and respiratory support modalities, and these patients were classified as the tracheostomy-positive group (tracheostomy [+]). For model training, the tracheostomy-negative group (tracheostomy [−]) was defined as patients who required uninterrupted IMV for five consecutive days from the time of ICU admission but did not undergo tracheostomy for any reason during the subsequent ICU stay.

### 2.5. Statistical Analysis

Continuous variables were summarized using median values with interquartile ranges (IQRs), whereas categorical variables were expressed as counts and percentages. Group comparisons were conducted using the Mann–Whitney U test for continuous data and the chi-square test for categorical data. Statistical significance was defined as a *p*-value below 0.05. AI–ML algorithms were employed to predict tracheostomy requirement.

Model performance was evaluated using discrimination metrics (AUROC and AUPRC) threshold-dependent metrics (accuracy, sensitivity, specificity, and F1-score), and calibration metrics (Brier score and calibration curve).

During model development, performance metrics—including discrimination, threshold-dependent measures, and Brier score—were internally assessed within the training dataset using 5-fold stratified cross-validation.

Final model performance, including ROC–AUC, AUPRC, accuracy, sensitivity, specificity, F1-score, calibration curve, calibration slope, and Brier score, was subsequently evaluated on the independent hold-out test dataset using predicted probabilities.

All analyses were conducted using Python version 3.10 software(Python Software Foundation, Wilmington, DE, USA)

## 3. Results

A total of 6507 patients who received IMV for at least five consecutive days were included in the analysis. Of these, 1049 patients (16.1%) underwent tracheostomy during their ICU stay, while 5458 patients (83.9%) were managed without tracheostomy. Baseline demographic characteristics were comparable between groups. The percentage of male patients was comparable between the tracheostomy and non-tracheostomy groups (61.6% vs. 59.1%, *p* = 0.122), and median age was similar (64.0 [50.8–74.0] vs. 63.0 [48.0–75.0] years, *p* = 0.323). Patients who underwent tracheostomy exhibited significantly lower ICU mortality compared with those who did not (31.8% vs. 38.7%, *p* < 0.001), a difference that was also evident in 28-day ICU mortality rates (23.8% vs. 36.5%, *p* < 0.001). Patients in the tracheostomy group demonstrated a significantly longer ICU length of stay compared with the non-tracheostomy group (445.5 [307.8–627.0] vs. 121.0 [47.0–280.8], *p* < 0.001). Mean arterial, systolic and diastolic blood pressure values were higher in the tracheostomy group, whereas HR and RR were lower (all *p* < 0.001). Oxygenation indices revealed lower OI and OSI values in the tracheostomy group, indicating relatively better oxygenation status (both *p* < 0.001). In contrast, ventilatory burden-related parameters, including work of breathing (WOB), VOX, PPeak, and PEEP, were significantly higher in patients who underwent tracheostomy (*p* < 0.001). Additionally, patients in the tracheostomy group had lower GCS scores and markedly higher secretion count compared with those who did not undergo tracheostomy (median secretion count: 11.0 [8.0–15.0] vs. 1.0 [1.0–3.0], *p* < 0.001). Inflammatory and metabolic parameters also differed between groups, with higher CRP levels and slightly higher BMI observed in the tracheostomy group (both *p* < 0.001), whereas arterial pH values were comparable between the groups (*p* > 0.05). Between-group comparisons demonstrated statistically significant differences across multiple variables. However, given the large sample size, small absolute differences between groups were frequently associated with statistically significant *p*-values (Table 1).

To further evaluate the contribution of clinical, physiological, and ventilatory parameters to tracheostomy requirement, ML-based analyses were subsequently performed. Table 2 presents the internal validation performance of the ML models obtained using 5-fold stratified cross-validation within the training dataset. Performance metrics are reported as mean ± standard deviation across the five folds. Overall, several models demonstrated good to excellent discrimination for predicting tracheostomy requirement, with ROC–AUC values ranging from 0.71 to 0.92. Gradient Boosting achieved the highest discriminative performance (AUC 0.92), followed by AdaBoost and Linear Discriminant Analysis and Random Forest models (AUC 0.91) and Logistic Regression (AUC 0.90). Multilayer Perceptron showed slightly lower performance (AUC 0.88), whereas Decision Tree exhibited substantially lower discrimination (AUC 0.71).

Despite comparable AUC values among several high-performing models, threshold-dependent metrics revealed notable differences in classification behavior. Most models achieved high specificity (≥0.95), whereas sensitivity was generally modest, ranging from 0.23 to 0.50 across models. Naïve Bayes represented an exception, demonstrating high sensitivity (0.86) at the expense of markedly reduced specificity (0.48) and overall accuracy.

Precision–recall analysis further highlighted differences in model performance in the context of class imbalance. AUPRC values were highest for Gradient Boosting and AdaBoost (0.58), followed closely by Random Forest (0.57), indicating superior precision–recall trade-offs compared with other algorithms. Models with similar ROC–AUC values therefore showed variable performance when evaluated using AUPRC and F1 score, underscoring the importance of complementary metrics beyond ROC analysis alone.

Based on clinical variables collected during the first five days of IMV following ICU admission, the Gradient Boosting model showed strong discriminative ability for predicting tracheostomy requirement (AUC 0.92, AUPRC 0.58), along with high specificity (95%), a balanced F1 score (0.51), and good calibration as reflected by a low Brier score (0.08). The Gradient Boosting model achieved the lowest Brier score among evaluated models (Table 2).

To evaluate model performance on unseen data, the trained models were assessed using the independent hold-out test dataset, which was not used during model development. The test set performance metrics are summarized in Table 3. On the independent test dataset, the Gradient Boosting model achieved an AUROC of 0.92 and an AUPRC of 0.56. Sensitivity was 0.36, specificity was 0.97, F1 score was 0.46 and the Brier score was 0.078. The negative predictive value was 0.89. Comparable AUROC values were observed for AdaBoost (0.92) and Logistic Regression (0.91), while other models demonstrated variable performance across threshold-dependent metrics (Table 3).

### Calibration Analysis of Model Performances

**Figure 2:**
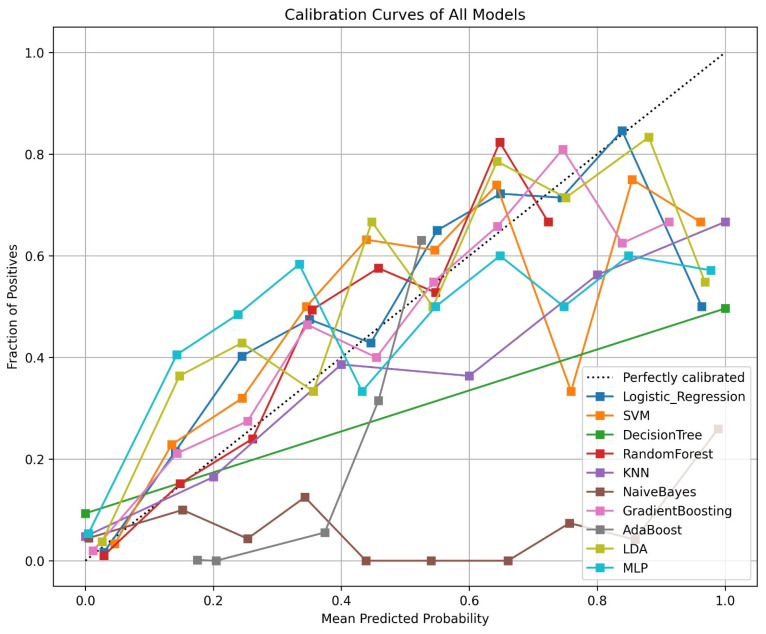
Calibration curves of machine learning models for predicting tracheostomy requirement. The dashed diagonal line represents perfect calibration, where predicted probabilities equal observed event frequencies. Abbreviations: SVM: Support Vector Machine, KNN: K-Nearest Neighbors, LDA: Linear Discriminant Analysis, MLP: Multilayer Perceptron.

Calibration slopes of ML models are shown in Figure 2.

**Figure 3:**
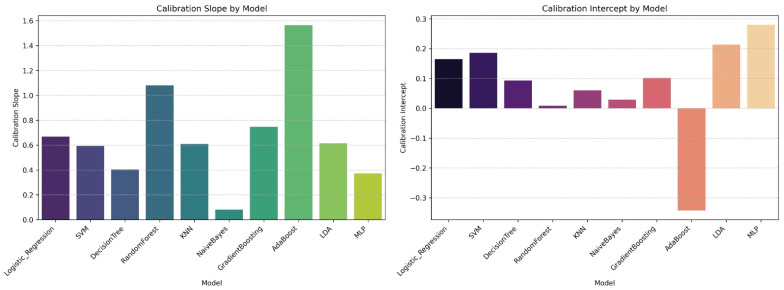
Calibration slope and intercept values across all machine learning models. Calibration performance of the machine learning models evaluated on the independent test dataset. The left panel shows the calibration slope and the right panel shows the calibration intercept for each model. A calibration slope of 1 and an intercept of 0 indicates perfect calibration. Abbreviations: KNN: K-Nearest Neighbors, LDA: Linear Discriminant Analysis, MLP: Multilayer Perceptron.

Calibration assessment across models demonstrated differences in calibration slope and intercept values. The Gradient Boosting model exhibited a calibration slope close to unity and an intercept near zero. In contrast, AdaBoost and Random Forest displayed larger deviations in both calibration slope and intercept (Figure 3).

Considering discrimination, calibration, and overall performance metrics collectively, the Gradient Boosting model was selected as the final model for subsequent Shapley Additive exPlanations (SHAP)-based interpretability and clinical evaluation.

**Figure 4:**
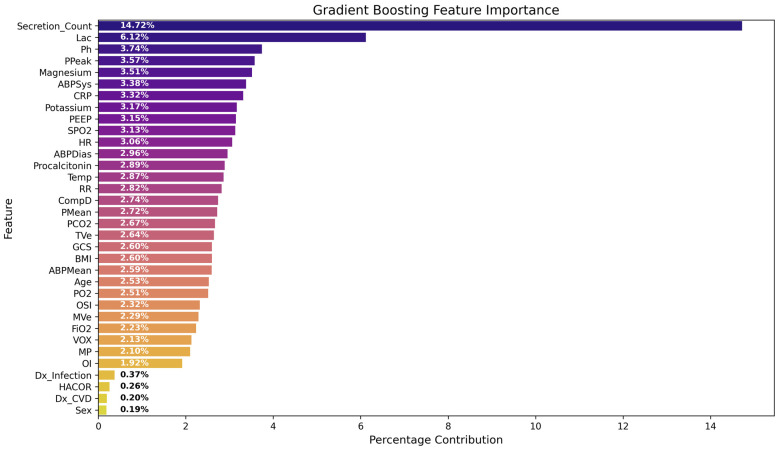
Feature importance of the Gradient Boosting model for predicting tracheostomy. The bars represent the relative contribution of each feature to the model prediction, expressed as percentage importance. Secretion count and lactate level were identified as the most influential predictors. Abbreviations: Lac: Lactate, PPeak: Peak inspiratory pressure, ABPSys: Systolic arterial blood pressure, CRP: C-reactive protein, PEEP: Positive end-expiratory pressure, SPO_2_: Peripheral oxygen saturation, HR: Heart rate, ABPDias: Diastolic arterial blood pressure, Temp: Temperature, RR: Respiratory rate, CompD: Dynamic compliance, Pmean: Mean airway pressure, PCO_2_: Partial pressure of carbon dioxide, TVe: Expiratory tidal volume, GCS: Glasgow Coma Scale, BMI: Body mass index, ABPMean: Mean arterial blood pressure, PO_2_: Partial pressure of oxygen, OSI: Oxygen saturation index, MVe: Expiratory minute ventilation, FiO_2_: Fraction of inspired oxygen, VOX: Ventilatory Oxygenation Index, MP: Mechanical Power, OI: Oxygenation index, Dx: Diagnosis, HACOR: Heart rate, Acidosis, Consciousness, Oxygenation, Respiratory rate Score, Dx_CVD: Cerebrovascular disease diagnosis.

Feature importance analysis of the Gradient Boosting model identified secretion count as the most influential predictor of tracheostomy requirement, accounting for approximately 14.72% of the total model contribution. This was followed by lactate level (6.12%), arterial pH (3.74%), PPeak (3.57%), and serum magnesium level (3.51%). Additional contributors included inflammatory markers, hemodynamic variables, and respiratory parameters such as PEEP, SpO_2_, RR and ventilatory pressures. GCS, gas exchange variables (PaO_2_, PaCO_2_, OI), and ventilatory mechanics (TVe, MP) showed moderate contributions to the model, whereas demographic variables and baseline comorbidities contributed minimally (Figure 4).

SHAP analysis of the Gradient Boosting model provided insight into the direction and magnitude of individual feature effects on tracheostomy prediction. Higher secretion count was consistently associated with an increased predicted probability of tracheostomy, whereas lower values were associated with a reduced predicted risk. Lower lactate levels similarly showed a positive contribution to the model output. Lower GCS scores were associated with higher predicted tracheostomy risk, while higher arterial pH values and increased airway pressures (including PPeak and PEEP) tended to shift predictions toward tracheostomy requirement. Hemodynamic variables and oxygenation-related parameters demonstrated mixed but clinically interpretable effects, with both positive and negative SHAP values observed across their ranges.

**Figure 5:**
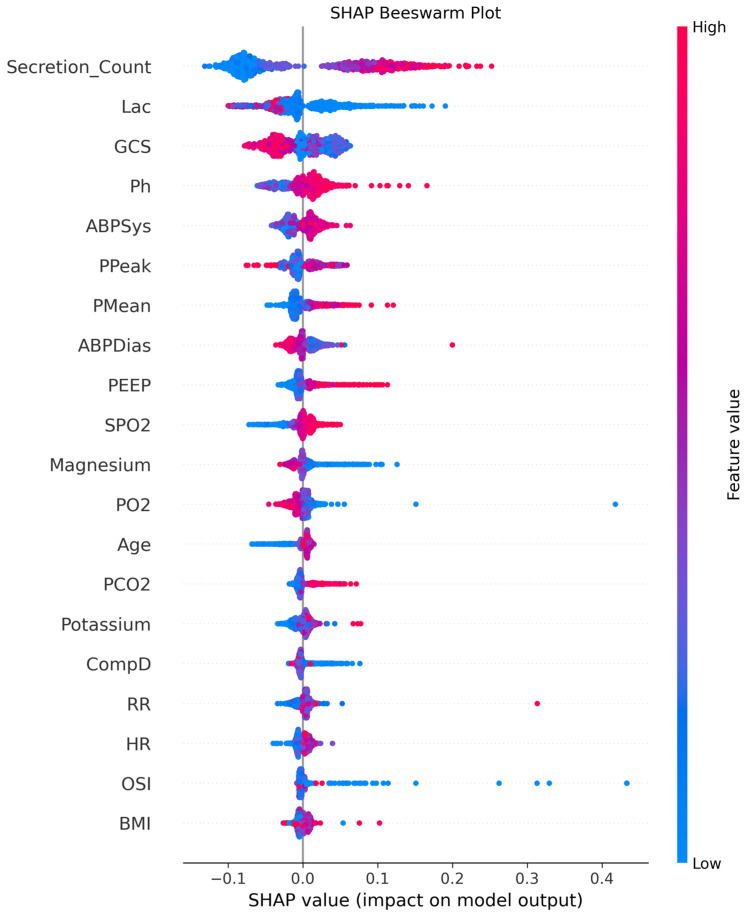
SHAP-based feature importance of the Gradient Boosting model for tracheostomy prediction. SHAP beeswarm plot illustrating the impact of each feature on the prediction of tracheostomy requirement in critically ill patients. Each dot represents an individual patient. The position on the x-axis indicates the SHAP value, reflecting the magnitude and direction of the feature’s contribution to the model prediction. Red points represent higher feature values and blue points represent lower feature values. Features are ranked according to their overall importance across the dataset. Abbreviations: Lac: Lactate, GCS: Glasgow Coma Scale, ABPSys: Systolic arterial blood pressure, PPeak: Peak inspiratory pressure, PMean: Mean airway pressure ABPDias: Diastolic arterial blood pressure, PEEP: Positive end-expiratory pressure, SPO_2_: Peripheral oxygen saturation, PO_2_: Partial pressure of oxygen, PCO_2_: Partial pressure of carbon dioxide, CompD: Dynamic compliance, RR: Respiratory rate, HR: Heart rate, OSI: Oxygen saturation index, BMI: Body mass index.

Overall, SHAP analysis confirmed that secretion count, metabolic derangement, neurological status, and ventilatory mechanics were the dominant contributors influencing model predictions, whereas demographic variables showed comparatively smaller effects on the predicted outcome (Figure 5).

## 4. Discussion

In this study, the predictive performance for identifying tracheostomy requirements was evaluated by integrating clinical and laboratory findings with MV parameters obtained during the first five days of IMV. The Gradient Boosting model demonstrated the most favorable overall performance for predicting tracheostomy requirement, achieving a strong balance between discrimination and calibration (AUC 0.92, AUPRC 0.56, specificity 97%, F1 score 0.46, Brier score 0.078). These findings suggest that ensemble-based boosting approaches may provide reliable probabilistic estimates in complex clinical decision-making scenarios such as tracheostomy prediction in critically ill patients. To enhance clinical interpretability beyond predictive accuracy, SHAP was applied alongside the Gradient Boosting model, allowing assessment of both the direction and relative importance of contributing features. This approach demonstrated that the model’s decision-making process was consistent with established clinical reasoning, supporting its potential utility as a clinical decision support tool. The findings indicate that tracheostomy decisions are inherently multifactorial, influenced by both airway management-related variables—such as secretion count, lactate, GCS, PPeak and laboratory parameters—reflecting systemic physiological stress. Taken together, these findings underscore the need to assess both airway-related and systemic factors simultaneously when evaluating critically ill patients for tracheostomy.

Tracheostomy is an ancient procedure with origins dating back to antiquity, and uncertainties remain regarding who first performed it and for what exact purpose. Descriptions of tracheostomy can be found in ancient Egyptian and Indian texts. In its earliest depictions, the procedure was described as perforation of the throat as a life-saving intervention in cases of choking or foreign body aspiration. As a formal surgical procedure, tracheostomy is reported to have been performed around 100 BC by the Greek physician Asclepiades, as later documented by Galen in the 2nd–3rd century AD [19]. Although uncertainties persist today regarding optimal timing, technique, and patient selection, the indication for tracheostomy has evolved beyond its historical role as an emergency airway intervention. In modern practice, particularly in critically ill patients, it has increasingly become an alternative strategy aimed at facilitating long-term ventilatory support and improving patient comfort and care in the ICU.

When making the decision to perform a tracheostomy, clinicians assess whether patients meet certain predefined clinical criteria. However, there is no universally applicable scale, score, or set of conditions that can be adapted to all patients for prediction. Existing scoring systems have primarily been developed for patients with intracerebral hemorrhage and traumatic cervical spinal cord injury [20,21,22]. However, each of these tools is applicable only to specific patient populations and demonstrates limited predictive power. As data availability expands and technological capabilities continue to evolve, ML methods enable the simultaneous evaluation of numerous variables to support prediction of this decision and to identify models or factors that offer the best predictive performance.

ML has increasingly been applied beyond critical care settings to support clinical decision-making in complex scenarios influenced by multivariable and dynamic physiological conditions. In cardiothoracic surgery, ML models have demonstrated utility in predicting postoperative complications and assisting clinicians in decision-making through preoperative risk stratification analyses [23,24]. Similarly, in thoracic surgery, the use of ML has expanded with the aim of improving intraoperative and postoperative outcomes [25]. The predictive success observed in these fields suggests that ML-based approaches may also provide meaningful clinical guidance in tracheostomy decision-making, a process influenced by dynamic and multifactorial variables.

ML has also been used in previous studies to predict weaning success from MV; however, studies focusing on the prediction of tracheostomy requirement remain limited. Prolonged ventilation and the requirement for tracheostomy varies depending on the patient, the underlying disease, and timing. Indeed, in the prospective validation of a model incorporating criteria such as intubation in the ICU, tachycardia, renal dysfunction, acidemia, elevated creatinine levels, and decreased bicarbonate levels to predict prolonged MV, the ML model outperformed existing scoring systems and demonstrated that patients requiring longer durations of MV met a greater number of these criteria [26]. Another study showed that for trauma patients, the strongest predictors of prolonged MV were the use of vasoactive agents, FiO_2_, PaCO_2_, PaO_2_, and the APACHE II score [27]. In the study by Yutaka et al., the most important variables were duration of MV, PaO_2_, blood urea nitrogen, HR and the GCS [28]. Unlike parameters traditionally associated with prolonged MV in prior studies, secretion count, lactate level, arterial pH, and PPeak emerged as more prominent predictors of tracheostomy requirement in our cohort. Interestingly, MP was lower in patients who subsequently required tracheostomy. This finding suggests that prolonged ventilatory dependence leading to tracheostomy may not necessarily be driven by higher mechanical ventilatory load but rather by factors such as impaired airway protection, secretion burden, neurological status, and prolonged weaning difficulties. Therefore, ventilatory intensity alone may not adequately capture the clinical complexity leading to tracheostomy decisions.

Predicting the need for tracheostomy may assist clinicians in the decision-making process by improving time and resource management. Following prolonged MV, the need for tracheostomy typically emerges after the first week [29,30]. For this reason, we aimed to predict which patients would require tracheostomy based on data from the first five days of IMV.

In mechanically ventilated trauma populations, numerous variables have been reported to predict the need for tracheostomy, and an analysis of 12 observational studies demonstrated that the presence of pneumonia was the most important determinant of tracheostomy requirement [12]. In another ML study predicting tracheostomy in prolonged MV, the most important parameter was the pulmonary component of the logistic organ dysfunction score [31]. In line with previously reported findings demonstrating an association between retained secretions and pneumonia, as well as between the development of pneumonia and tracheostomy, secretion burden emerged as the most heavily weighted parameter in predicting the need for tracheostomy [32]. Based on the current literature, prior ML studies predicting tracheostomy have not incorporated secretion-related variables. In contrast, our study demonstrated that the count of secretion aspiration outperformed all other clinical, laboratory, and disease severity scores in predicting tracheostomy. This finding is also consistent with one of the established indications for tracheostomy—facilitation of secretion drainage. It is well recognized that severe secretion retention is associated with prolonged tracheostomy [33].

Another important predictive parameter was a low GCS score. Evidence from patients with traumatic brain injury indicates that individuals presenting with a GCS score of 4 or lower on the fifth postoperative day after decompressive craniectomy subsequently underwent tracheostomy in all reported cases [34]. Furthermore, studies have identified both a low GCS score and an elevated rapid shallow breathing index as key predictors of tracheostomy requirement in patients with traumatic spinal cord injury [35]. In the present study, the comparatively lower contribution of GCS within the Gradient Boosting model may reflect the confounding influence of sedative medications on accurate neurological assessment. Notably, SHAP-based interpretation indicates that a low GCS remains an important determinant in tracheostomy decision-making; however, this discrepancy highlights a limitation of the analysis and underscores the need for complementary or additional modeling approaches. Furthermore, patients with brain death were not excluded from the cohort, and some patients with low GCS scores may have experienced mortality without requiring tracheostomy. This heterogeneity may have further complicated the Gradient Boosting analysis and attenuated the apparent importance of GCS.

The strong predictive weight of secretion count suggests that longitudinal airway management variables may be more informative than the others. Instead, all tracheostomies performed after the first five days of IMV during the ICU stay were included, provided that they were secondary to prolonged MV. This approach was deliberately chosen to exclude patients admitted to the ICU for planned tracheostomy and to focus on prolonged MV. By capturing tracheostomies occurring throughout the ICU stay, this strategy minimized bias related to non-patient-related factors, such as institutional practices or staffing constraints, and enabled a more accurate reflection of clinically driven decision-making.

Predicting outcomes over limited time frames is difficult. In a ML model based on the first-day parameters of traumatic brain injury patients admitted to ICU, the model with the highest performance had an AUC of 0.66 [36]. In patients with sepsis admitted to the ICU, the STeP model—developed using data from the first 24 h to predict tracheostomy within 14 days—achieved an AUC of 0.73 in the validation cohort [37]. Among ML models developed to predict tracheostomy using admission-day variables, the pruned XGBoost model demonstrated a specificity of 0.957 and an AUROC of 0.794. In this pruned XGBoost model, the most important parameters for predicting tracheostomy were the presence of chronic respiratory failure, septic shock, and pneumonia [38]. When ICU admission data were used, the proposed ML model demonstrated good discriminatory performance in predicting MV duration, with an accuracy of 79.1% and an AUROC of 0.82. SHAP analysis revealed that the most influential parameters for predicting the duration of MV were tracheostomy status, type of ICU admission, PaO_2_, and pH [39]. Previous studies have generally focused on limited time windows—particularly admission-day parameters—when developing ML models for prediction. Therefore, we aimed to extend our analysis to a longer observation period by incorporating a 5-day clinical course, allowing our model to evaluate a broader temporal window and consequently achieve higher specificity. In determining the need for tracheostomy, not only baseline characteristics at admission but also clinically important variables that evolve during the course of illness may influence decision-making.

When we evaluated the association between tracheostomy requirement and disease categories, infectious and cerebrovascular disease groups emerged as the most heavily weighted disease classes in the Gradient Boosting model. Similarly, in a study conducted in Japan, respiratory and neurological diseases were the disease groups most frequently requiring tracheostomy [40]. Another study demonstrated that elevated lactate levels are associated with an increased need for MV [41]. In the present study, interpretability analyses indicated that lower lactate concentrations were linked to a higher probability of tracheostomy, a result that may, at first, seem unexpected. However, this pattern likely reflects the fact that patients with lower lactate levels have a greater probability of survival and clinical stabilization, thereby reaching a stage at which tracheostomy becomes a relevant therapeutic consideration. In contrast, patients with severe metabolic derangement and elevated lactate levels may experience early mortality, precluding tracheostomy.

The first strength of this study, the use of a heterogeneous dataset that includes patients with multiple comorbidities, a wide range of parameters, and a broad temporal distribution—while integrating clinically meaningful variables together with established scoring systems—enhances diversity and supports the development of more generalizable predictions. This study notably incorporates both clinical status and relevant parameters obtained during the first five days of IMV in the ICU to estimate tracheostomy need.

The high specificity provided by the classification model may be particularly useful for optimizing resource allocation and supporting early clinical decision-making, potentially helping to reduce complications associated with prolonged MV. This study also demonstrates that, although prediction models may achieve high learning accuracy and specificity, sensitivity—and consequently the F1 score—may not always reach comparable levels, underscoring the need to interpret all predictors collectively rather than in isolation. From a clinical implementation perspective, the interpretable SHAP-based approach helps clinicians understand the relative importance of specific variables and may facilitate the adoption of reliable and explainable AI in routine clinical practice.

Previous studies differ in terms of the techniques used, datasets, selected variables, and timing of assessments, which makes direct comparison of models challenging. Early clinical prediction of tracheostomy requirement by intensive care physicians remains limited. Therefore, ML models hold significant potential for successfully predicting the need for tracheostomy in the future.

First, of the limitations of this study, the model was developed from a single center in Türkiye; therefore, multicenter external validation will be essential to assess its clinical utility. Although the classification model may perform well within this dataset, its generalizability to different patient populations—varying in demographics, comorbidities, or institutional protocols—may be limited. Nevertheless, the inclusion of a Turkish ICU dataset highlights the critical importance of developing models tailored to the unique challenges and characteristics of healthcare systems in middle-income countries. Tracheostomy decisions were based on multidisciplinary clinical assessment; however, variability in physician judgment and institution-specific practice patterns cannot be fully excluded. As this was a single-center retrospective study, clinical decision-making may have been influenced by local protocols, resource considerations, or physician preference, potentially introducing bias unrelated to strict patient-level necessity. Importantly, patients were classified according to tracheostomy performed rather than independently adjudicated medical necessity. Therefore, variability in clinical judgment may have influenced outcome classification. Multicenter external validation studies are required to confirm the robustness and generalizability of the proposed model across diverse institutional settings. Although our analysis incorporated a large number of parameters and scoring systems, some potentially influential variables may have been omitted. Finally, while the current model predicts the need for tracheostomy, it does not address the equally important issue of optimal timing, which remains an area for future development and improvement.

## 5. Conclusions

The model underscores that tracheostomy indication is not driven by a single parameter but rather by the combined influence of clinical status, respiratory mechanics, neurological status, and metabolic stress. The SHAP analysis demonstrated that the model predicts tracheostomy decisions in a clinically coherent manner. The relationships between these parameters and tracheostomy need highlight that tracheostomy timing should be guided by an integrated assessment of respiratory and systemic physiological burden. AI-supported prediction models are being increasingly integrated into clinical practice. Future research may enable the development of a novel scoring system derived from multiple parameters. The use of multicenter datasets derived from improved electronic health record systems, together with alternative modeling approaches and analyses across different patient subgroups, is expected to enhance external validity, individualized prediction, and overall generalizability. Although external validation is required, the proposed model demonstrates the potential of early clinical and ventilatory data to support individualized tracheostomy decision-making in critically ill patients.

## Figures and Tables

**Table 1:** Baseline characteristics of patients with and without tracheostomy.

Variable	Tracheostomy (+)	Tracheostomy (−)	*p*-Value
	*n*: 1049	*n*: 5458	
Gender (male)	646 (61.6%)	3223 (59.1%)	0.122
ICU mortality	333 (31.8%)	2114 (38.7%)	<0.001
ICU mortality at 28 days	249 (23.8%)	1993 (36.5%)	<0.001
Age	64.0 (50.8–74.0)	63.0 (48.0–75.0)	0.323
HACOR	8.0 (8.0–8.0)	8.0 (8.0–10.0)	<0.001
ABPMean	80.8 (76.3–85.5)	78.5 (69.7–86.7)	<0.001
ICU_LOS (h)	445.5 (307.8–627.0)	121.0 (47.0–280.8)	<0.001
PBW	65.9 (56.0–70.5)	65.9 (56.0–70.5)	0.055
HR	83.9 (73.0–95.4)	89.8 (78.2–103.4)	<0.001
SPO_2_	96.4 (94.7–97.8)	96.2 (93.8–97.8)	<0.001
OI	486.7 (332.7–722.7)	529.9 (390.6–780.4)	<0.001
VOX	1.0 (0.6–1.4)	0.3 (0.2–0.4)	<0.001
WOB	1.1 (1.0–1.3)	1.0 (0.8–1.2)	<0.001
Temp	36.4 (35.9–36.9)	36.5 (36.0–36.9)	0.019
PCO_2_	45.3 (40.3–51.9)	42.2 (36.8–49.7)	<0.001
Secretion count	11.0 (8.0–15.0)	1.0 (1.0–3.0)	<0.001
ABPDias	60.1 (54.4–65.5)	58.7 (51.2–66.1)	<0.001
OSI	513.7 (399.3–680.3)	524.4 (447.1–668.1)	<0.001
BMI	26.9 (24.5–30.1)	26.0 (23.9–27.8)	<0.001
PMean	11.7 (10.2–13.9)	11.8 (11.0–13.3)	<0.001
RR	15.5 (14.5–17.0)	16.4 (14.5–20.0)	<0.001
PO_2_	104.1 (87.4–123.9)	99.0 (75.3–123.1)	<0.001
GCS	7.0 (5.0–8.0)	8.0 (5.0–12.0)	<0.001
PEEP	7.0 (5.9–8.1)	6.2 (5.4–7.5)	<0.001
CompD	38.6 (31.4–46.8)	41.4 (32.3–53.5)	<0.001
MVe	7.4 (6.6–8.2)	7.9 (6.7–9.2)	<0.001
MP	4.6 (3.4–6)	11.2 (9.3–13.5)	<0.001
pH	7.4 (7.3–7.4)	7.4 (7.3–7.5)	0.093
CRP	109.1 (31.0–174.5)	82.0 (35.0–131.0)	<0.001
ABPSys	126.9 (117.8–136.7)	119.0 (104.3–131.3)	<0.001
PPeak	20.7 (18.2–23.3)	19.8 (17.5–22.7)	<0.001

Abbreviations: Data are presented as median (interquartile range, IQR) for continuous variables and as number (percentage) for categorical variables. Comparisons between groups were performed using the Mann–Whitney U test for continuous variables and the chi-square test for categorical variables. A ***p***-value < 0.05 was considered statistically significant. ICU: Intensive care unit, ABPMean: Mean arterial blood pressure, LOS: Length of stay, PBW: Predicted body weight, HR: Heart rate, OI: Oxygenation index, VOX: Volume oxygenation index, WOB: Work of breathing, Temp: Temperature, ABPDias: Diastolic arterial blood pressure, OSI: Oxygen saturation index, BMI: Body mass index, RR: Respiratory rate, GCS: Glasgow Coma Scale, CompD: Dynamic compliance, MVe: Expiratory minute ventilation, MP: Mechanical power, CRP: C-reactive protein, ABPSys: Systolic arterial blood pressure, PPeak: Peak pressure.

**Table 2:** Internal validation performance metrics of machine learning models using 5-fold cross-validation (training set).

Model	Accuracy (Mean ± SD)	AUC (Mean ± SD)	AUPRC (Mean ± SD)	Sensitivity (Mean ± SD)	Specificity (Mean ± SD)	F1 Score (Mean ± SD)	Brier Score
Logistic Regression	0.88 ± 0.01	0.90 ± 0.01	0.54 ± 0.04	0.35 ± 0.03	0.97 ± 0.01	0.45 ± 0.04	0.09
AdaBoost	0.88 ± 0.01	0.91 ± 0.01	0.58 ± 0.02	0.40 ± 0.07	0.96 ± 0.01	0.48 ± 0.05	0.11
Gradient Boosting	0.88 ± 0.01	0.92 ± 0.01	0.58 ± 0.05	0.45 ± 0.02	0.95 ± 0.01	0.51 ± 0.02	0.08
LDA	0.88 ± 0.01	0.91 ± 0.00	0.55 ± 0.04	0.34 ± 0.03	0.97 ± 0.00	0.44 ± 0.03	0.09
SVM	0.88 ± 0.01	0.88 ± 0.01	0.54 ± 0.04	0.27 ± 0.03	0.98 ± 0.00	0.38 ± 0.03	
Random Forest	0.87 ± 0.01	0.91 ± 0.00	0.57 ± 0.04	0.23 ± 0.04	0.98 ± 0.00	0.34 ± 0.05	0.08
MLP	0.86 ± 0.01	0.88 ± 0.01	0.49 ± 0.05	0.45 ± 0.07	0.93 ± 0.01	0.47 ± 0.05	0.11
Decision Tree	0.85 ± 0.01	0.71 ± 0.02	0.52 ± 0.03	0.50 ± 0.03	0.91 ± 0.01	0.49 ± 0.03	0.14
KNN	0.85 ± 0.01	0.78 ± 0.02	0.38 ± 0.03	0.25 ± 0.03	0.95 ± 0.00	0.32 ± 0.04	0.11
Naive Bayes	0.54 ± 0.07	0.79 ± 0.02	0.39 ± 0.04	0.86 ± 0.02	0.48 ± 0.08	0.34 ± 0.03	0.39

Abbreviations: Values are presented as mean ± standard deviation across five cross-validation folds. LDA: Linear Discriminant Analysis, SVM: Support Vector Machine, MLP: Multilayer Perceptron, KNN: K-Nearest Neighbors, AUC: Area Under the Receiver Operating Characteristic Curve, AUPRC: Area Under the Precision–Recall Curve, SD: Standard Deviation.

**Table 3:** Performance of machine learning models evaluated on the independent test dataset.

Model	F1 Score	Sensitivity	Specificity	NPV	AUROC	AUPRC	Brier Score
Decision Tree	0.46	0.43	0.93	0.91	0.68	0.51	0.145
AdaBoost	0.46	0.36	0.97	0.90	0.92	0.57	0.110
Gradient Boosting	0.46	0.36	0.97	0.89	0.92	0.56	0.078
Logistic Regression	0.45	0.34	0.97	0.89	0.91	0.54	0.088
MLP	0.43	0.36	0.94	0.89	0.90	0.52	0.106
LDA	0.43	0.36	0.94	0.89	0.91	0.55	0.091
SVM	0.39	0.28	0.98	0.89	0.89	0.54	0.090
KNN	0.33	0.24	0.97	0.88	0.90	0.52	0.109
Random Forest	0.31	0.22	0.98	0.88	0.91	0.57	0.081
Naive Bayes	0.28	0.20	0.96	0.88	0.89	0.46	0.391

Abbreviations: Performance metrics were calculated using predicted probabilities obtained from the independent test set, which was strictly held out during model development. MLP: Multilayer Perceptron, LDA: Linear Discriminant Analysis, SVM: Support Vector Machine, KNN: K-Nearest Neighbors, NPV: Negative Predictive Value, AUROC: Area Under the Receiver Operating Characteristic Curve, AUPRC: Area Under the Precision–Recall Curve.

## Data Availability

De-identified patient data supporting the findings of this study are available from the corresponding author upon reasonable request. Access to the data is subject to approval by the ICU chief of Bakırkoy Dr. Sadi Konuk Training and Research Hospital, in accordance with local regulations on the protection of patient data.

## Abbreviations

ABPDias	Diastolic Arterial Blood Pressure
ABPMean	Mean Arterial Blood Pressure
ABPSys	Systolic Arterial Blood Pressure
AI	Artificial Intelligence
AUC	Area Under the Curve
AUPRC	Area Under the Precision–Recall Curve
APACHEII	Acute Physiology and Chronic Health Evaluation II
BMI	Body Mass Index
CRP	C-Reactive Protein
CompD	Dynamic Lung Compliance
ΔP	Driving Pressure
FiO_2_	Fraction of Inspired Oxygen
GCS	Glasgow Coma Scale
HACOR	Heart Rate, Acidosis, Consciousness, Oxygenation, Respiratory Rate Score
HR	Heart Rate
ICU	Intensive Care Unit
IMV	Invasive Mechanical Ventilation
LOS	Length of Stay
ML	Machine Learning
MP	Mechanical Power
MV	Mechanical Ventilation
MVe	Minute Ventilation
OI	Oxygenation Index
OSI	Oxygen Saturation Index
PaCO_2_	Partial Pressure of Arterial Carbon Dioxide
PaO_2_	Partial Pressure of Arterial Oxygen
PBW	Predicted Body Weight
PEEP	Positive End-Expiratory Pressure
PPeak	Peak Inspiratory Pressure
PMean	Mean Airway Pressure
ROC	Receiver Operating Characteristic
RR	Respiratory Rate
SHAP	SHapley Additive exPlanations
SOFA	Sequential Organ Failure Assessment
SpO_2_	Peripheral Oxygen Saturation
VOX	Volume Oxygenation Index
VT	Tidal Volume
WOB	Work of Breathing
